# The pathogenicity and future treatment strategies of *Candida albicans*

**DOI:** 10.3389/fcimb.2026.1752304

**Published:** 2026-02-19

**Authors:** Jiadi Wu, Chenyang Jiang, Hui Wang, Tongbin Chen, Xi Chen, Wenyue Da

**Affiliations:** 1 Department of Pathology, The Third Affiliated Hospital of Soochow University, Changzhou, Jiangsu, China; 2 Guangzhou Institute of Cancer Research, Affiliated Cancer Hospital of Guangzhou Medical University, Guangzhou, China

**Keywords:** biofilm, *Candida albicans*, drug resistance, host immunity, novel antifungal therapy

## Abstract

*Candida albicans* (*C. albicans*) is a major pathogenic fungus that severely impacts on human health. This review systematically elaborates on the key pathogenic processes of *C. albicans*, starting with its colonization, morphological transformation and biofilm formation under different carbon sources. The interaction between *C. albicans* and host immunity, including the role of PRRs, host genetics and immune polymorphisms, and trained immunity. Candidalysin regulating cAMP/PKA signaling pathway of *C. albicans* hyphae-biofilm transformation, the interaction between *C. albicans* and bacteria, as well as mucosal and invasive *C. albicans* infections, persister cells in anti-*C. albicans* therapy, emerging biology and pathogenicity aspects, epigenetic and chromatin regulation of host-drug adaptation, and strain-specific heterogeneity in pathogenicity, biofilm traits and drug susceptibility. Additionally, it summarizes novel therapeutic strategies, emphasizing probiotics and antimicrobial peptides (AMPs), and combination strategies with novel targeted therapy and traditional anti-fungal therapy to improve the survival of patients with *Candida albicans* infection. It systematically and comprehensively summarizes the pathogenicity of *C. albicans* and the possible therapeutic targets, providing new ideas for the development of novel antifungal drugs in the future.

## Introduction

*Candida albicans* (*C. albicans*) is usually a commensal organism found in the gastrointestinal tract, reproductive tract, oral cavity and skin of healthy individuals (d'Enfert et al., 2021; Gunsalus et al., 2016). In recent years, fungal infections have become increasingly common, especially among patients with cancer, those infected with the human immunodeficiency virus, or those whose immune systems have been weakened due to the use of immunosuppressive drugs (Benedict et al., 2019; Chang et al., 2017). Due to the disruption of the epithelial barrier and the dysfunction of the host immune system, *C. albicans* shifts from a symbiotic state to a pathogenic state (Lee et al., 2021; Lopes and Lionakis, 2022), possibly moving from the intestinal tract to the bloodstream (Wang, 2015), causing human diseases (d'Enfert et al., 2021; Pappas et al., 2016). The transition from yeast form to hyphal form is a key factor for the pathogenicity of *C. albicans* (Gow et al., 2011). Studies have found that when *C. albicans* grows as a symbiotic organism, both yeast and hyphal forms exist simultaneously throughout the entire intestinal tract (Witchley et al., 2019). This indicates that the expression of hyphal-specific virulence factors is the determining factor for *C. albicans* to transform from a symbiotic state to a pathogenic state (Witchley et al., 2019). The genes of *C. albicans* encode the cell wall proteins necessary for initial adhesion, colonization, and resistance to immune attack (Navarro-Garcia et al., 2001). In addition, they also encode various positive or negative regulatory factors related to pathogenicity, including RBF1, EFG1, and TUP1, as well as genes encoding secreted aspartic acid proteases involved in invading the host endothelial barrier and macrophage phagocytosis (Navarro-Garcia et al., 2001). *C. albicans* can exhibit different lifestyles to adapt to the constantly changing environmental conditions within the host. This change in the lifestyle of *C. albicans* is also controlled by various regulatory networks (Rai et al., 2021). Global epidemiological data confirm that *C. albicans* remains the most prevalent etiological agent of candidiasis, encompassing both mucosal and invasive infections across diverse clinical settings. specifically, studies have demonstrated that *C. albicans* accounts for the highest proportion of candidemia cases, serving as the leading pathogen in 92% of instances and maintaining its status as the most prevalent pathogenic species across various geographical regions (Guinea, 2014). Beyond its high epidemiological prevalence, *C. albicans* possesses unique pathogenic characteristics, such as robust morphological plasticity, efficient biofilm formation, and intricate interactions with the host immune system that distinguish it from most non-*C. albicans* species and are key contributors to its high virulence and clinical recalcitrance (Mayer et al., 2013). These pathogenic traits, in turn, give rise to specific clinical challenges associated with *C. albicans* infections, including its association with higher mortality rates in invasive cases and the emergence of drug resistance, collectively rendering it a critical priority for clinical and translational research (Soriano et al., 2023).

## Colonization of *C. albicans*

The colonization of fungi on medical implant materials has had a significant adverse impact on the healthcare industry, leading to the spread of hospital-acquired diseases and causing malfunctions of medical equipment (Harding and Reynolds, 2014). Various medical devices, including central venous catheters, urinary catheters, hip and knee implants, and dentures, have been contaminated with white candida biofilms (Lohse et al., 2018). Mainly because *C. albicans* can regulate the wetting property of its cell surface according to the materials on which the cells are attached (Murat et al., 2019). In fact, depending on the surrounding environment, *C. albicans* may express either hydrophilic or hydrophobic surface proteins (Singleton and Hazen, 2004). The hydrophilic fibrous proteins on the surface of *C. albicans* cells help to exhibit hydrophilicity (Hazen and Hazen, 1992), while the hydrophobicity of the cell surface is mainly determined by Csh1p (Hazen, 2004). Hydrophobic and hydrophilic cells have similar biochemical properties, but they have different cell wall structures at the ultrastructural level (Le et al., 2024). In the yeast form, *C. albicans* exhibits hydrophilic characteristics. When it transforms from yeast to hyphal form, the cell wall becomes hydrophobic, and finally forms a completely hydrophobic biofilm (Le et al., 2024). Hydrophobic *C. albicans* is more toxic than its hydrophilic counterpart (Danchik and Casadevall, 2020).

## C. albicans biofilms

*C. albicans* is a polymorphic yeast-like fungus. Depending on its environment, it undergoes morphological transitions from yeast-like, pseudohyphal, and hyphal cell types, and forms biofilms (Mayer et al., 2013). It is enveloped by an extracellular matrix composed of β-1,3 glucan, β-1,6 glucan, and α-1,2-branched α-1,6 mannan (Chandra et al., 2001). The study of microorganisms was based on suspension cultures or colonies growing on nutrient agar plates. However, it is now widely recognized that biofilms are the most ideal and possibly “natural” growth state for most microorganisms (Nobile and Johnson, 2015; Kolter and Greenberg, 2006). Biofilms are a group of microbial cells that are attached to a solid surface or exist at the liquid-gas interface, surrounded by an extracellular matrix, and possess characteristics different from those in a free suspension state (Nobile and Johnson, 2015; Kolter and Greenberg, 2006). Biofilms are an important pathogenic factor of *C. albicans*, forming a physical barrier to resist host immune factors and developing resistance to antifungal drugs (Pereira et al., 2021). The formation process of *C. albicans* biofilm is divided into four stages (Gulati and Nobile, 2016): 1.) Spreading stage: *C. albicans* attaches to the solid surface. 2.) Proliferation and early growth: Attached cells proliferate and begin to grow hyphae. 3.) Maturation stage: A mature biofilm is formed, consisting of hyphal cells, pseudohyphal cells and round yeast cells, forming a complex network, wrapped by the extracellular matrix, presenting a thick and clear structure. 4.) Dispersal stage: The biofilm gradually disperses into cell clusters mainly composed of yeast-like cells that have split from the hyphae, spreading to new locations to promote the spread of infection. The biofilm structure formed by *C. albicans* is extremely complex, it consists of yeast-like cells, pseudohyphal cells, and hyphal cells surrounded by the extracellular matrix (Chandra et al., 2001; Fox and Nobile, 2012). In addition to forming biofilms on implanted medical devices (such as catheters, pacemakers, heart valves, joint prostheses, and dentures), *C. albicans* biofilms can also form on host surfaces, including mucosal surfaces, epithelial cell layers, and parenchymal organs (Kojic and Darouiche, 2004). In fact, *C. albicans* is one of the most frequently isolated species associated with neonatal candidiasis, osteoarticular infections and endogenous endophthalmitis (Pappas et al., 2016). Additionally, more than 70% of denture stomatitis is related to microbial biofilm colonization on the acrylic base of dentures (Patil et al., 2015; Bajunaid et al., 2022). Colonization and biofilm formation of *C. albicans* are considered the main causes of inflammatory disease processes such as peri-implant mucositis, which will lead to implant failure (Souza et al., 2022).

The potential transcriptional regulatory network that controls the formation of *C. albicans* biofilms is the core mechanism for regulating the development and pathogenicity of biofilms. This network precisely drives the progress of each stage of biofilm formation and affects its drug resistance through the cooperative interaction, dynamic regulation, and specific signal integration of core transcription factors. Among them, the core transcription factor network composed of Efg1, Tec1, Bcr1, Ndt80, Rob1, and Brg1 is the key for regulation. On the one hand, the redundancy and genetic interaction of these core transcription factors reveal their dose-dependent regulatory mechanism and the core role in biofilm formation, providing a genetic basis for developing antifungal strategies targeting this network (Glazier et al., 2017). On the other hand, this recently evolved transcriptional network can precisely regulate the entire process of biofilm formation through the coordinated binding of downstream target genes, and its interaction mode and the specificity of target gene selection reflect the adaptive evolution characteristics of this network, providing a core mechanism framework for understanding the pathogenicity and drug resistance of *C. albicans* (Nobile et al., 2012). In addition, the special regulatory characteristics of transcription factors also participate in the regulation of biofilm formation, for example, the phase separation ability of network transcription factors can precisely regulate the expression of downstream virulence-related genes, thereby directly affecting the formation of hyphae and biofilm generation processes (Ganser et al., 2023). It is worth noting that the Ume6 protein complex, as a key node of the specific regulatory pathway, can integrate the regulation of genes related to morphogenesis, adhesion, and hypoxia response, through the formation of dynamic complexes with different cofactors, coordinating hyphal growth, cell adhesion, and hypoxia adaptability, thereby shaping the spatial structure of the biofilm and enhancing its drug resistance and pathogenicity. This also provides a new target for targeting the Ume6 complex to intervene in biofilm-related infections (Do et al., 2025).

## *C. albicans* biofilms and drug resistance

The existing antifungal drugs, when they can effectively act on the concentration of free-phase *C. albicans*, often have poor effects on the *C. albicans* biofilms. The biofilm structure confers robust resistance to conventional antifungals by impeding drug penetration, inducing metabolic dormancy and driving drug-resistance gene expression, leading to persistent and recurrent infections (Pereira et al., 2021). The biofilm matrix acts as a physical barrier to block antifungal penetration, and the sessile fungal cells exhibit metabolic dormancy and upregulated expression of drug efflux pumps and target-modifying genes, and the presence of drug-resistant cells are some of the main factors leading to immune evasion and weakened response to available drugs (Sahoo et al., 2025). Additionally, biofilm-associated stress responses and genetic heterogeneity further enhance cross-resistance to azoles, echinocandins and polyenes (Kaur and Nobile, 2023). Although higher concentrations of drugs can be used to combat the biofilms, these doses often cause serious side effects to the host. The resistance of antifungal drugs related to *C. albicans* biofilms and the colonization ability on implanted medical devices are associated with the increase in medical costs and poor patient prognosis (Lebeaux et al., 2014; Tumbarello et al., 2007). In the disseminated infection model of mice, yeast cells dispersed within mature biofilms have been proven to have higher pathogenicity (Matsubara et al., 2016). Given the limited treatment options, when local biofilm infection of the device is suspected, it is usually recommended to remove the medical device rather than to administer antifungal therapy (Pappas et al., 2009). The existence of such highly resistant biofilms poses a significant threat and requires attention.

## Drug tolerance and persister cells in Anti-*C. albicans* therapy

**Figure 1:**
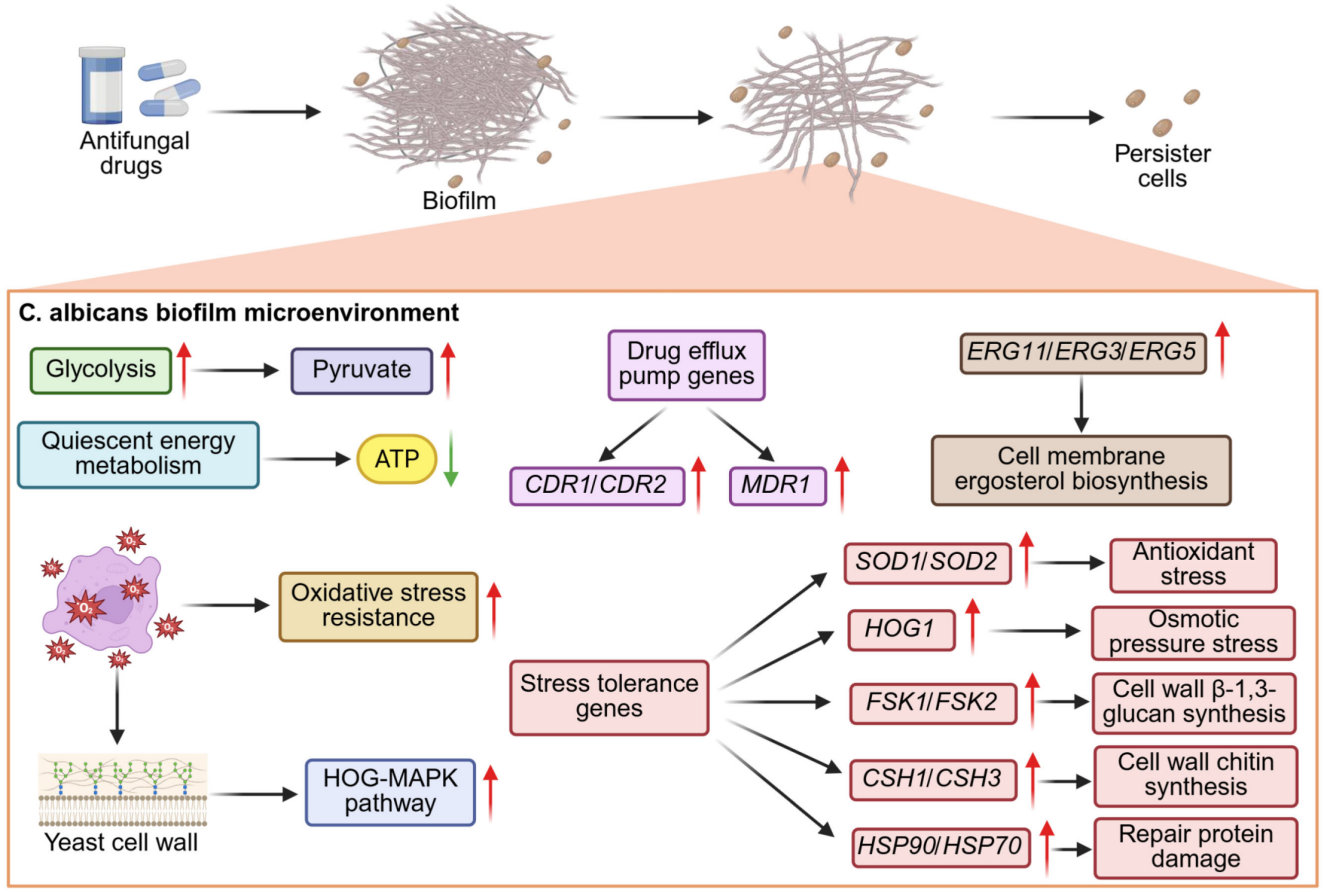
Antifungal drugs disrupt the biofilm of *C. albicans*, there exist antifungal-tolerant persistent cells. This is mainly due to the hypoxic biofilm microenvironment inducing oxidative stress, which leads to a decrease in the glycolysis of *C. albicans*, an enhancement of antioxidant stress, and quiescent energy metabolism. Antifungal drugs damage the cell wall of *C. albicans*, activate the HOG-MAPK pathway and the cell wall integrity pathway, triggering the stress defense response of the fungus. In addition, the expression of ERG family, drug efflux pump genes, and stress tolerance genes is upregulated, ultimately promoting the expansion of the highly resistant persisting subpopulation.

Antifungal-tolerant persister cells as a distinct subpopulation within *C. albicans* biofilms, which can survive lethal concentrations of multiple antifungal agents (fluconazole, amphotericin B, caspofungin) and are absent in planktonic fungal cultures. These persister cells exhibit a reversible, non-genetic phenotypic trait unrelated to heritable drug resistance mutations, with their progeny restoring wild-type antifungal susceptibility, and the biofilm-specific niche is confirmed to be essential for their formation (LaFleur et al., 2006). The biofilm matrix forms a physical-chemical barrier to reduce antifungal penetration and induce hypoxic/nutrient-limited niches, while *C. albicans* initiates metabolic reprogramming (e.g., suppressed glycolysis, enhanced oxidative stress resistance, and quiescent energy metabolism) in this niche to enter a dormant state, which directly mediates the formation and survival of antifungal-tolerant persister cells (Denega et al., 2019). *C. albicans* biofilm microenvironment further creates a hypoxic, nutrient-limited niche with high extracellular matrix deposition, which induces metabolic reprogramming and epigenetic remodeling in *C. albicans*, driving fungal cells into a metabolically quiescent state to form antifungal-tolerant persister cells that enable persistent infection and recurrent vulvovaginal candidiasis (RVVC). These findings confirm the critical role of biofilm and persister cells in the pathogenesis of vaginal candidiasis, providing novel targets for the development of anti-biofilm and anti-persister therapeutic strategies against this disease (Wu et al., 2020a). Studies have shown that caspofungin at sub-inhibitory concentrations significantly promotes the formation of persister cells in C. albicans, rather than inhibiting fungal survival or virulence traits. Firstly, the sub-inhibitory concentration of caspofungin induces damage stress in the cell wall of *C. albicans*, activates the HOG-MAPK pathway and the cell wall integrity pathway, triggering the stress defense response of the fungus, laying the phenotypic foundation for the formation of persisting cells. Secondly, under drug stress, the fungus undergoes core metabolic remodeling, with the activities of glycolysis and the tricarboxylic acid cycle being downregulated, and the cells entering a low metabolism and non-proliferative dormant state, significantly reducing their sensitivity to lethal doses of antifungal drugs. Thirdly, sub-inhibitory drug exposure can significantly upregulate the expression of ERG family, drug efflux pump genes, and stress tolerance genes in *C. albicans*, synergistically enhancing the drug tolerance of the fungus, and ultimately promoting the expansion of the highly resistant persisting subpopulation (**Figure 1**) (Ye et al., 2022). These findings highlight a critical clinical risk of sub-inhibitory echinocandin exposure, which may facilitate persister cells development and further lead to refractory *C. albicans* infections and treatment failure.

## Mucosal and invasive *C. albicans* infections

*C. albicans* infection exhibits distinct clinical manifestations and progression patterns that are tightly linked to mucosal and invasive infection stratification, as well as significant heterogeneity among different patient populations with variable immune status and clinical conditions. *C. albicans* achieves stable commensal colonization on healthy oral, esophageal, vulvovaginal and other mucosal epithelial surfaces by regulating the expression of adhesins and virulence factors, adapting to the nutrient-poor microenvironment of mucosae, and evading host immune surveillance with the synergistic effect of the resident microbiota that competes for nutrients and restricts its overgrowth (Schille et al., 2025). Meanwhile, the local mucosal barrier damage, microbial dysbiosis or host mild immune suppression can trigger the transition of *C. albicans* to pathogenicity, which is characterized by activated hyphal growth, enhanced expression of invasive virulence factors (e.g., candidalysin), and induction of epithelial cell injury and pro-inflammatory immune responses, thus leading to localized mucosal infections with mild clinical symptoms and favorable prognosis (Moyes et al., 2016). In cases of severe host immunosuppression or iatrogenic factors, *C. albicans* infection leads to a significant reduction in mucosal bacterial diversity, accompanied by the abnormal overgrowth of *Enterococcus* on oral and small intestinal mucosae, and the overproliferated *Enterococcus* can degrade the epithelial junction protein E-cadherin *in vitro* and *in vivo*, destroy the integrity of the mucosal epithelial barrier and increase its permeability. *C. albicans* can further break through the mucosal barrier to cause bloodstream dissemination and deep organ invasion, resulting in life-threatening invasive infections with high mortality (Bertolini et al., 2019). Invasive candidiasis is a severe and life-threatening healthcare-associated invasive fungal infection mainly caused by *C. albicans* and an increasing number of non*-C. albicans* species (e.g., *Candida glabrata*, *Candida parapsilosis* and the multidrug-resistant *Candida auris*), which primarily arises from the host endogenous fungal colonization and is triggered by impaired host immune defenses and damaged mucosal/epithelial barriers; it manifests as a spectrum of clinical conditions dominated by candidemia, and can further develop into disseminated infections involving multiple deep organs such as the kidneys, liver and heart valves, with non-specific clinical symptoms and an attributable mortality rate of approximately 30% (Pappas et al., 2018). Recent study has shown that the tripartite crosstalk of the host, *C. albicans* and vaginal microbiota in VVC and RVVC, focusing on the core mechanisms of fungal commensal-to-pathogen transition and infection development in the vaginal niche. Healthy vaginal microbiota dominated by *Lactobacillus* species confers critical protection against *C. albicans* pathogenicity via nutrient competition and acidic microenvironment maintenance, while microbiota dysbiosis and fungal virulence factor secretion drive fungal overgrowth, vaginal epithelial damage and inflammatory responses that induce VVC. Probiotic supplementation and microbiome restoration offer new insights into the treatment of vaginal candidiasis (Valentine et al., 2025).

## Key factors of the host microenvironment regulate the pathogenicity of *C. albicans*

The host microenvironment factors related to the characteristics of *C. albicans*, such as the level of CO_2_ and the state of hypoxia, have been proven to significantly affect the metabolism, morphology and pathogenicity of this fungus; at the same time, the competition for glucose resources in the microenvironment also profoundly regulates the interaction between *C. albicans* and the host and the outcome of infection. At the level of glucose metabolism, the host’s glucose homeostasis is a key guarantee for the survival of immune cells during *C. albicans* infection. *C. albicans* competes with host immune cells for glucose in the microenvironment, and maintaining glucose homeostasis can enhance the antifungal function of immune cells, thereby improving the survival rate of the host in systemic fungal infections (Tucey et al., 2018). In anoxic environment, *C. albicans* can induce a “masking” phenomenon on its surface β-glucan through the mitochondrial and cAMP-protein kinase A signaling pathways, thereby avoiding recognition and attack by host immune cells (such as macrophages), significantly enhancing its immune evasion ability and pathogenic survival potential in the host (Pradhan et al., 2018). Under physiological concentration of CO_2_ conditions, the fungus can comprehensively enhance the attachment, maturation and dispersion stages of biofilms through the Ras/cAMP/PKA signaling pathway and core regulatory factors such as Efg1. At the same time, it improves the ability to absorb iron/glucose and develops resistance to azoles, helping it efficiently adapts to the host’s nutrient-limited microenvironment, and further strengthening its pathogenicity (Pentland et al., 2021).

## Emerging new aspects of *C. albicans* biology and pathogenicity

In recent years, mounting studies have unveiled novel biological aspects underlying the virulence of *C. albicans*, which extend far beyond the classic virulence factors. Three critical and emerging regulatory axes have been identified as core determinants of *C. albicans* pathogenicity and host adaptation, including epigenetic regulation of virulence traits, metabolic plasticity coupled with host-fungal metabolic crosstalk, and the impact of symbiotic microbial interactions on fungal pathogenicity. First, epigenetic regulation of virulence traits has emerged as a core adaptive mechanism, histone H3 lysine 56 acetylation (H3K56ac) in regulating the pathogenicity of *C. albicans*, H3K56ac directly modulates the transcription of hypha-specific genes (*ECE1*, *HWP1*) and phenotypic switching. H3K56 acetylation as a master epigenetic switch linking chromatin dynamics to *C. albicans* virulence, providing a novel target for developing antifungal therapies that disrupt fungal adaptive strategies (Conte et al., 2024). Second, metabolic plasticity and host–fungal metabolic crosstalk are now recognized as key pathogenicity determinants. After recognizing *C. albicans*, macrophages will activate aerobic glycolysis to provide the energy needed for the immune response. *C. albicans* switches its metabolic mode, mainly relying on glycolysis in the hypoxic mucosal environment and oxidative phosphorylation in the oxygen-rich environment, adapting to different microenvironments of the host and consuming glucose to weaken the function of macrophages (Pellon et al., 2022). *C. albicans* relies on multiple alternative carbon pathways to survive macrophage phagosomes, enhance stress/antifungal resistance, and drive virulence, showing these pathways act as both nutrient sources and environmental signals (Williams and Lorenz, 2020). Third, the influence of symbiotic bacteria on the pathogenicity of *C. albicans*. *Lactobacillus crispatus* protects vaginal epithelial cells from *C. albicans* by reducing fungal adhesion/invasion and reshaping innate immune cytokine secretion, highlighting its role in vaginal microecological defense against VVC (Niu et al., 2017).

## Epigenetic and chromatin regulation of *C. albicans* adaptive plasticity to host and drugs

Epigenetic regulation and chromatin modification are critical drivers of the rapid adaptive plasticity of *C. albicans* to host microenvironments and antifungal drug pressure, and exert profound impacts on fungal pathogenicity. Histone acetyltransferases (HATs) and histone deacetylases (HDACs) perform essential functions relating to growth, virulence, drug resistance and stress responses of *C. albicans* (Su et al., 2020). Histone post-translational modifications represented by H3K56ac are core epigenetic regulatory modes in *C. albicans*. Hst3p, a key sirtuin histone deacetylase, mediates H3K56 deacetylation to maintain chromatin repression, and its inhibition leads to genome-wide H3K56ac enrichment, which further dysregulates the transcription of virulence-associated genes including adhesins, phenotypic switching regulators and hyphal formation-related factors, thereby disrupting yeast-hyphae transition, white-opaque switching and host adhesion processes essential for *C. albicans* infection (Conte et al., 2022). The histone deacetylase Sir2 is a key virulence regulator of *C. albicans* during systemic infection, as it enhances the fungus’s immune escape ability by mediating cell wall remodeling to reduce host immune recognition, and sustains the metabolic activity critical for fungal survival and colonization in host tissues, ultimately promoting the progression of systemic *C. albicans* infection and boosting fungal pathogenicity *in vivo* (Yang et al., 2024). In addition, the pericentromeric chromatin of *C. albicans* exhibits a unique hybrid epigenetic feature with characteristics of both euchromatin and heterochromatin, which not only ensures the stability and normal function of centromeres during fungal cell division, but also regulates genome plasticity and expression of adjacent virulence-related genes, thereby playing an essential role in mediating the adaptability and pathogenicity of *C. albicans* in host microenvironments (Freire-Beneitez et al., 2016). Chromatin remodeling and epigenetic reprogramming enable *C. albicans* to rapidly adjust its gene expression profile without genetic mutations, which not only enhances fungal adaptability to dynamic host niches and immune stress, but also modulates fungal drug tolerance and the formation of persister cells, ultimately affecting the pathogenicity, infection persistence and therapeutic efficacy of *C. albicans*.

## Metal ion limitation and carbon source differences

In the host microenvironment, the regulation of key metal ions such as iron and zinc mediated by nutritional immunity, as well as the differences in carbon sources, jointly constitute the core nutritional signals that control the commensal-pathogenic transformation of *C. albicans*. *C. albicans*, by precisely sensing this signal and initiating dynamic strategies for nutrient acquisition and metabolic remodeling, becomes a crucial guarantee for its colonization of the host and pathogenic invasion. In an iron-limited environment, *C. albicans* can finely regulate multiple iron uptake pathways such as high-affinity iron reduction systems and heme uptake through mechanisms such as the Sef1-Sfu1-Hap43 regulatory circuit and the iron-sulfur cluster assembly system, thereby promoting invasive and morphological transformation processes related to virulence, and accelerating the progression of infection (Zheng et al., 2025). At the same time, it also possesses a highly flexible high-affinity iron transport system that can switch different uptake strategies by sensing changes in host microenvironment iron concentrations, adapting to both commensal and pathogenic states (Mamouei et al., 2017). It is worth noting that under iron starvation conditions, *C. albicans* will also initiate carbon metabolism remodeling independent of SEF1. Iron limitation inhibits iron-sulfur cluster-dependent protein lipoylation, causing the loss and inactivation of the LAT1 subunit of pyruvate dehydrogenase, thereby promoting glucose flow to the pentose phosphate pathway to increase NADPH production, and inducing non-PDH pathways such as the pyruvate bypass and fatty acid oxidation to synthesize acetyl coenzyme A, thereby maintaining survival in an iron-limited environment (Garg et al., 2025). At the zinc-limited level, host immune cells secrete calprotectin, which can create a “metal ion starvation” state by chelating zinc ions and copper ions, forming an antifungal defense barrier, restricting nutrient acquisition by *C. albicans* and inhibiting its growth and virulence (Besold et al., 2018). *C. albicans* can adaptively respond to this stress through phenotypic regulation-zinc ion limitation induces its “giant adhesion phenotype”, which significantly enhances its adhesion ability to host cells and biofilm formation, thereby increasing its colonization and pathogenic potential under nutrient-deficient conditions (Malavia et al., 2017). Apart from the limitation of metal ions, the differences in carbon sources also drive adaptive changes in *C. albicans*, manifested as significant differences in growth rate, cell adhesion ability, cell wall structure, biofilm formation efficiency, and hyphal formation ability. These changes will further affect its drug resistance. The above carbon source-dependent phenotypes and physiological alterations directly or indirectly regulate the pathogenicity of *C. albicans* in the host environment.

In order to cause infection in the host body, *C. albicans* needs to absorb locally available carbon sources in order to grow, divide and occupy various habitats (Lok et al., 2021). *C. albicans* usually inhabits environments with limited glucose supply but rich in alternative carbon sources (Lok et al., 2021). It may have evolved the ability to absorb multiple carbon sources simultaneously, which promotes its growth and pathogenicity (Brown et al., 2007). When growing on different carbon sources, *C. albicans* exhibits different morphological characteristics. Cells cultured on glucose mainly exhibit hyphal growth, while they grow in yeast form under lactate culture conditions (Alves et al., 2017). Different carbon sources also affect the growth of *C. albicans*, which grows faster in glucose-containing medium than in galactose-containing medium (Jin et al., 2004). Studies have found that the presence or absence of glucose does not affect the carbon assimilation of *C. albicans* when alternative carbon sources are present, which helps this fungus to exert pathogenic effects in various nutrient-limited host microenvironments (Sandai et al., 2012). When *C. albicans* is phagocytosed by macrophages, JEN1 and JEN2 are upregulated, indicating that these genes help *C. albicans* to utilize lactate in the phagosome of macrophages and survive in the environment lacking glucose after phagocytosis (Lok et al., 2021).

The cell wall of *C. albicans* is an indispensable structure. The cell wall is involved in adhesion, colonization, signal transduction and immune recognition, and plays an important role in the infection process because its dissolution leads to cell rupture and death. Therefore, it is the main target of antifungal drugs (Arana et al., 2009). Different carbon sources affect the glucan structure in the cell wall of *C. albicans*. The surface details of cells growing on glucose, cotton sugar, inositol and rhamnose are relatively rough, while those growing on galactose, maltose, sucrose, fructose, xylose and glycogen are relatively smooth (Lok et al., 2021). Compared with cells cultured in glucose, the cross-linking degree of β-1,6 glucan in *C. albicans* cultured in lactic acid is lower, which reduces the hardness of the cell wall (Pemmaraju et al., 2016). The biofilm of *C. albicans* is highly pathogenic and its formation varies in different carbon sources. The biofilm formed by cells cultured with sucrose has a higher average roughness, height and thickness, followed by those formed by cells cultured with glucose, arabinose and lactic acid (Pemmaraju et al., 2016). Compared with cells growing in glucose, *C. albicans* grown in lactic acid shows a stronger ability to form biofilm (Alves et al., 2017). Cells grown under lactic acid conditions may form biofilm more efficiently than those grown under glucose conditions (Ene et al., 2012a).

Under the influence of different carbon sources, the yeast-hyphal transformation of *C. albicans* varies. In the cells of *C. albicans* cultivated on a specific medium with glucose as the carbon source, the production of pseudofilaments has increased, and it is even higher when cultured on galactose, xylose, or rhamnose (Lok et al., 2021). Indeed, studies have confirmed that low glucose concentrations can induce the hyphal development of *C. albicans* (Vidotto et al., 1996). It was found that N-acetylglucosamine can promote the transformation of *C. albicans* from budding yeast to hyphal growth (Alvarez and Konopka, 2007; Naseem et al., 2017). Compared with glucose, the cells of *C. albicans* cultivated in N-acetylglucosamine show better performance in triggering the formation of hyphal tubes (Lok et al., 2021).

Within the host, differences in carbon sources can significantly affect the cytotoxicity of *C. albicans*. In systemic and vaginal infection mouse models, compared with mice infected with cells cultured only with glucose, mice infected with *C. albicans* cultured with lactic acid, glucose plus lactic acid, or amino acid mixtures had a higher fungal load and more significant weight loss in their bodies (Ene et al., 2012b). Compared with cells grown under glucose culture conditions, *C. albicans* grown under lactic acid culture conditions inhibits the immune response of human macrophages, with lower levels of IL-6 and TNF-α. Under lactic acid culture conditions, as well as in the mixed culture of glucose and lactic acid, the efficiency of *C. albicans* being phagocytosed by macrophages is lower, indicating that *C. albicans* grown under lactic acid culture conditions is more toxic than that grown under glucose culture conditions (**Table 1**) (Ene et al., 2013). Additionally, compared with cells grown in glucose culture medium, *C. albicans* grown in lactic acid culture medium has stronger resistance to antifungal drugs caspofungin and amphotericin B, as well as the antibiotic tunicamycin (Ene et al., 2012b).

**Table 1:** The influence of *C. albicans* under different carbon sources.

Characteristic	Glucose	Galactose	Sucrose	Lactate	Reference
Morphological characteristics	Hypha	Hypha	–	Yeast	(Lok et al., 2021; Alves et al., 2017)
Growth	Fast	Slow	–	–	(Jin et al., 2004)
Cell wall	Rough	Smooth	Smooth	–	(Lok et al., 2021)
Biofilm	Thin	–	Thick	Rather thick	(Alves et al., 2017; Pemmaraju et al., 2016; Ene et al., 2012a)
Cytotoxicity	Weak	–	–	Strong	(Ene et al., 2013)

In summary, in-depth analysis of the synergistic adaptation mechanism of *C. albicans* to metal ion limitations in the host environment and carbon source differences is of great significance for the development of more effective preventive measures and treatment strategies to cope with *C. albicans* infections in the future.

## *C. albicans*/PRRs

The interaction between *C. albicans* and the host immune system is pivotal in maintaining the symbiotic state and driving the infection process (Wang, 2015). The host’s innate immune defense against *C. albicans* is particularly dependent on pattern recognition receptors (PRRs) (Zheng et al., 2015), which are expressed on various cell types and recognize conserved pathogen-associated molecular patterns (PAMPs) of *C. albicans*, including Toll-like receptors (TLRs), C-type lectin receptors (CLRs), and nucleotide-binding oligomerization domain (NOD)-like receptors (NLRs).

As the first line of defense, epithelial cells utilize specific PRRs to recognize *C. albicans* and initiate early defensive responses. While the exact PRR repertoire in epithelial cells is less extensively characterized compared to professional immune cells, emerging evidence indicates that CLRs (e.g., Dectin-1) and TLRs are involved in sensing *C. albicans* at epithelial surfaces. This initial recognition not only triggers epithelial cell-autonomous responses (e.g., barrier reinforcement, secretion of proinflammatory cytokines) but also primes the subsequent recruitment and activation of innate immune cells.

In contrast, innate immune cells (neutrophils, monocytes/macrophages, dendritic cells (DCs)) express a more diverse set of PRRs that mediate robust antifungal responses. Among these, CLRs play a central and synergistic role. For instance, Dectin-1, a key CLR, recognizes β-glucan in the *C. albicans* cell wall and triggers a cascade of cellular responses, including activation of the nuclear transcription factors NF-κB and IRF5, the ERK-MAPK pathway, the NLRP3 inflammasome, as well as phagocytosis and respiratory burst (Kimberg and Brown, 2008). Importantly, Dectin-1 can also cooperate with TLRs to fine-tune the antifungal immune response. Functional studies have further confirmed the criticality of CLR synergy: mice with double deletion of Dectin-1 and Dectin-2, or triple deletion of Dectin-1, Dectin-2, and Mincle, exhibit significantly increased susceptibility to systemic *C. albicans* infection. These multiple CLR-knockout mice fail to control fungal growth due to inadequate early responses mediated by inflammatory monocytes, highlighting that the cooperative action of CLRs is essential for effectively regulating systemic *C. albicans* infection and preserving organ function (Thompson et al., 2019).

TLRs, another major class of PRRs highly expressed on macrophages, neutrophils, and DCs, also contribute to antifungal immunity by recognizing distinct *C. albicans* cell wall components: TLR2 and TLR4 sense phospholipid mannan and O-mannan, respectively (Bao et al., 2023). Activation of these TLRs triggers the NF-κB and mitogen-activated protein kinase (MAPK) pathways, leading to the production of proinflammatory cytokines (TNF-α, IL-6, IL-1) and chemokines (CXCL-1, CXCL-2) (Wang et al., 2019). These soluble factors play a crucial role in recruiting and activating additional immune cells to the infection site, thereby enhancing the clearance of pathogenic fungi.

Beyond their role in host defense, the interactions between *C. albicans* PAMPs and PRRs also have broader implications for host physiology and disease. For example, excessive alcohol consumption promotes the translocation of intestinal β-glucan to the liver, where it binds to Dectin-1 on liver macrophages, activating the NLRP3 inflammasome and contributing to the development of alcoholic hepatitis (Wu et al., 2020b). Conversely, fungal exosomes hold promise as vaccines: their specific components (e.g., mannan, glucan) can interact with TLR4 and Dectin-1 on immune cells, offering a potential strategy for combating or preventing candidiasis (Honorato et al., 2024).

## *C. albicans* and host immunity

The synergistic relationship between fungal infections and tumor immunity has garnered growing attention, with *C. albicans* being identified as a specific fungus closely associated with tumorigenesis (Gu and Jia, 2024). *C. albicans* establishes a symbiotic relationship with the host through complex immune evasion mechanisms and pathogenic processes linked to morphological changes. Under conditions of microbial homeostasis, *C. albicans* contributes to maintaining immune balance, however, its dysregulation can lead to severe mucosal infections, systemic infections, tumorigenesis, and neurological diseases (Gu and Jia, 2024). A comprehensive understanding of the host immune pathways activated by C. albicans and the corresponding fungal evasion strategies is critical for deciphering this complex host-pathogen interaction, and key molecules in these pathways (e.g., TBK1, cGAS-STING, PD-L1) play pivotal roles in regulating immune responses.

First, the innate immune response is initiated upon the detection of pathogen-associated molecular patterns (PAMPs) from *C. albicans*, which triggers the activation of TANK-binding kinase 1 (TBK1)—a serine/threonine kinase central to innate immune signaling cascades that regulates the production of type I interferons (IFN-I) and proinflammatory cytokines (Perry et al., 2005). Specifically, TBK1 exerts its regulatory function through two major pathways: the TBK1-IRF3 pathway, which primarily drives IFN-I production, and TBK1-related NF-κB pathways, which are key for cytokine secretion. In the context of anti-*C. albicans* immunity, macrophages activate the TBK1-IRF3 pathway via the interaction between InsP3R (inositol 1,4,5-trisphosphate receptor) and SEC5 on phagosomes; this activation initiates the IFN-I response, thereby modulating the host’s antifungal immune defense (Yang et al., 2018). Notably, pathogens have evolved sophisticated strategies to exploit TBK1 for immune evasion (Gu and Jia, 2024). The effector protein Cmi1 secreted by *C. albicans* translocates to the host cytoplasm and binds to TBK1, disrupting TBK1-mediated phosphorylation of IRF3. This interference inhibits the IFN-I signaling cascade in macrophages, ultimately suppressing the host immune response (Luo et al., 2024). Beyond fungal infections, TBK1 is also implicated in tumor immune evasion by reducing the sensitivity of tumor cells to TNF-α and IFN-γ, thereby dampening anti-tumor immunity (Sun et al., 2023a). This dual role of TBK1 in fungal and tumor immune evasion underscores its potential as a critical cross-talk node between fungal infections and tumor immunity.

Another key immune regulatory axis involved in *C. albicans*-host interactions is the cGAS-STING-TBK1 pathway. The cGAS-STING pathway is a major cytosolic DNA-sensing pathway, where cGAS detects foreign DNA and synthesizes cyclic GMP-AMP, which then binds to and activates STING. Activated STING recruits and activates TBK1, thereby initiating IFN signaling (Cheng et al., 2020). This pathway activation leads to increased expression of PD-L1 inhibits T-cell activation and promotes immune evasion by binding to PD-1 on T cells. In *C. albicans* infections, PD-L1 is specifically involved in fungal immune evasion by suppressing the release of neutrophils from bone marrow reserves to peripheral tissues, thereby impairing the host’s ability to clear the fungus (Yu et al., 2022). Additionally, recent studies have highlighted the regulatory role of STING in antifungal immunity: STING deficiency enhances the antifungal immune response during systemic *C. albicans* infections. Targeting the interaction between STING and Src using the STING N18 peptide inhibits Syk-mediated signal transduction and strengthens the host’s antifungal response, suggesting promising applications for developing antifungal therapeutics (Chen et al., 2023).

Apart from the TBK1 and cGAS-STING pathways, other host immune molecules also modulate anti-*C. albicans* responses. For instance, the C-type lectin receptor CLEC2D inhibits IRF5 activation through dimerization, thereby reducing the IL-12-driven antifungal effect against *C. albicans* (Li et al., 2023). The E3 ubiquitin ligase Casitas B-cell lymphoma protein b (Cbl-b) weakens antifungal immunity by promoting the ubiquitination and degradation of Dectin-2 and Dectin-3—key C-type lectin receptors that recognize *C. albicans* cell wall components (Zhu et al., 2016). In addition to immune evasion and host defense mechanisms, *C. albicans* colonization also plays a protective role in maintaining host immune homeostasis. Intestinal colonization by *C. albicans* is crucial for sustaining systemic antifungal Th17 immunity, as it activates the immune system to accumulate CD4+ T cells that secrete protective IL-17, thereby preventing invasive fungal infections (Shao et al., 2019). Moreover, MUC2—the major mucin in the intestinal mucus layer—inhibits the yeast-to-hypha transition of *C. albicans*, a key morphological change associated with fungal pathogenicity (Takagi et al., 2022).

## Host genetics and immune polymorphisms in *Candida* infection

Host genetics and immune polymorphisms represent critical determinants of inter-individual variability in susceptibility to *C. albicans* infection and therapeutic responses, with the Dectin-1/CARD9/IL-17 signaling pathways emerging as the most clinically relevant axes. When the cell wall components such as β-glucan of *C. albicans* are recognized by Dectin-1, they activate NF-κB and IRF through the Syk/CARD9/Bcl10/MALT1 axis, driving the secretion of IL-1β, IL-6, and IL-23, thereby inducing the production of IL-17A/F and IL-22 by Th17 cells, recruiting neutrophils and enhancing the epithelial barrier, forming the core anti-fungal defense line (Swidergall, 2019). In terms of mucosal infection susceptibility, the Dectin-1 Y238X heterozygote significantly increases the risk of RVVC, and is associated with a higher *Candida* colonization rate in patients with hematological malignancies, requiring more frequent prophylactic antifungal treatment (Rosentul et al., 2011). Patients with pure CARD9 deficiency often present with Familial chronic cutaneous and mucosal candidiasis (CMC), accompanied by stubborn infections in the skin, nails, and mouth, and some may have deep fungal diseases, making conventional treatment difficult to cure (Glocker et al., 2009). Defects in the IL-17 pathway are an important cause of early-onset CMC. In patients with such defects, the neutrophil chemotaxis and killing ability are decreased, making them prone to recurrent infections (Puel et al., 2012). In terms of systemic infection and prognosis, although the polymorphism of Dectin-1/CARD9 is not consistently associated with the susceptibility to *Candida* bloodstream infection, the responses of the heterozygous Y238X carriers to cytokines *in vitro* are weakened, which may affect the inflammatory regulation and prognosis of severe infections (Rosentul et al., 2011). CARD9-deficient patients have invasive infections, the mortality rate significantly increases, and they are prone to atypical site infections. CARD9-deficient patients have limited efficacy to conventional antifungal drugs such as azoles and echinocandins, and require prolonged treatment courses or combined immunomodulatory therapy. In addition to eliminating the pathogen with conventional antifungal drugs, for those with pathway defects, recombinant IL-17, granulocyte-macrophage colony-stimulating factor (GM-CSF), or thymosin, etc. are combined for use to enhance the immune effect. For central nervous system infections caused by the CARD9 defect, a combination of surgical drainage and long-term antifungal treatment is required (Glocker et al., 2009). Therefore, Dectin-1 agonists or β-glucan vaccines can enhance Th17 responses, providing a new approach for infection prevention in high-risk populations (Shen et al., 2020).

## Trained immunity in against *C. albicans*

The trained immunity is driven by epigenetic reprogramming and metabolic rewiring of innate immune cells upon primary stimulation with microbial components, cytokines or other stimuli. This reprogramming endows innate cells with a long-term, enhanced functional phenotype, enabling them to mount more robust and rapid pro-inflammatory, anti-infective and tissue repair responses against subsequent heterogeneous pathogenic challenges (Netea et al., 2016). The study demonstrates that mTOR/HIF-1α axis-driven aerobic glycolysis serves as the fundamental metabolic basis of trained immunity. Activation of mTOR and upregulation of HIF-1α trigger enhanced glycolytic reprogramming in innate immune cells, which fulfills the elevated metabolic demands of trained immunity and synergizes with epigenetic remodeling to sustain the long-term pro-inflammatory and anti-pathogen memory phenotype of innate cells (Cheng et al., 2014). Glutathione synthesis as a critical priming regulator of β-glucan-induced trained immunity in monocytes. It demonstrates that glutathione production drives metabolic reprogramming and epigenetic modifications in monocytes, which are essential for the induction of enhanced anti-infective phenotypes characteristic of trained immunity. This finding uncovers a novel molecular link between glutathione metabolism and innate immune memory, highlighting its key role in mediating protective responses against pathogens such as *C. albicans* (Su et al., 2021). Recent study reports the development of a novel protein-free vaccine that effectively stimulates innate immune responses and confers robust protection against multiple nosocomial pathogens, including *C. albicans* and drug-resistant bacteria. The vaccine exerts its protective effects by triggering trained immunity via metabolic and epigenetic reprogramming of innate immune cells, without relying on adaptive immune responses induced by protein antigens. This innovative protein-free design not only overcomes the limitations of traditional protein-based vaccines but also provides a promising strategy for combating life-threatening nosocomial infections and addressing the global challenge of antimicrobial resistance (Yan et al., 2023). Overall, trained immunity exerts a pivotal protective effect against *C. albicans* infection. Trained immunity can reverse immune tolerance to *C. albicans* in immunocompromised hosts and improve anti-fungal immune defense capacity, which also provides a novel direction for the development of anti-*Candida* preventive strategies and vaccine research.

## Candidalysin

**Figure 2:**
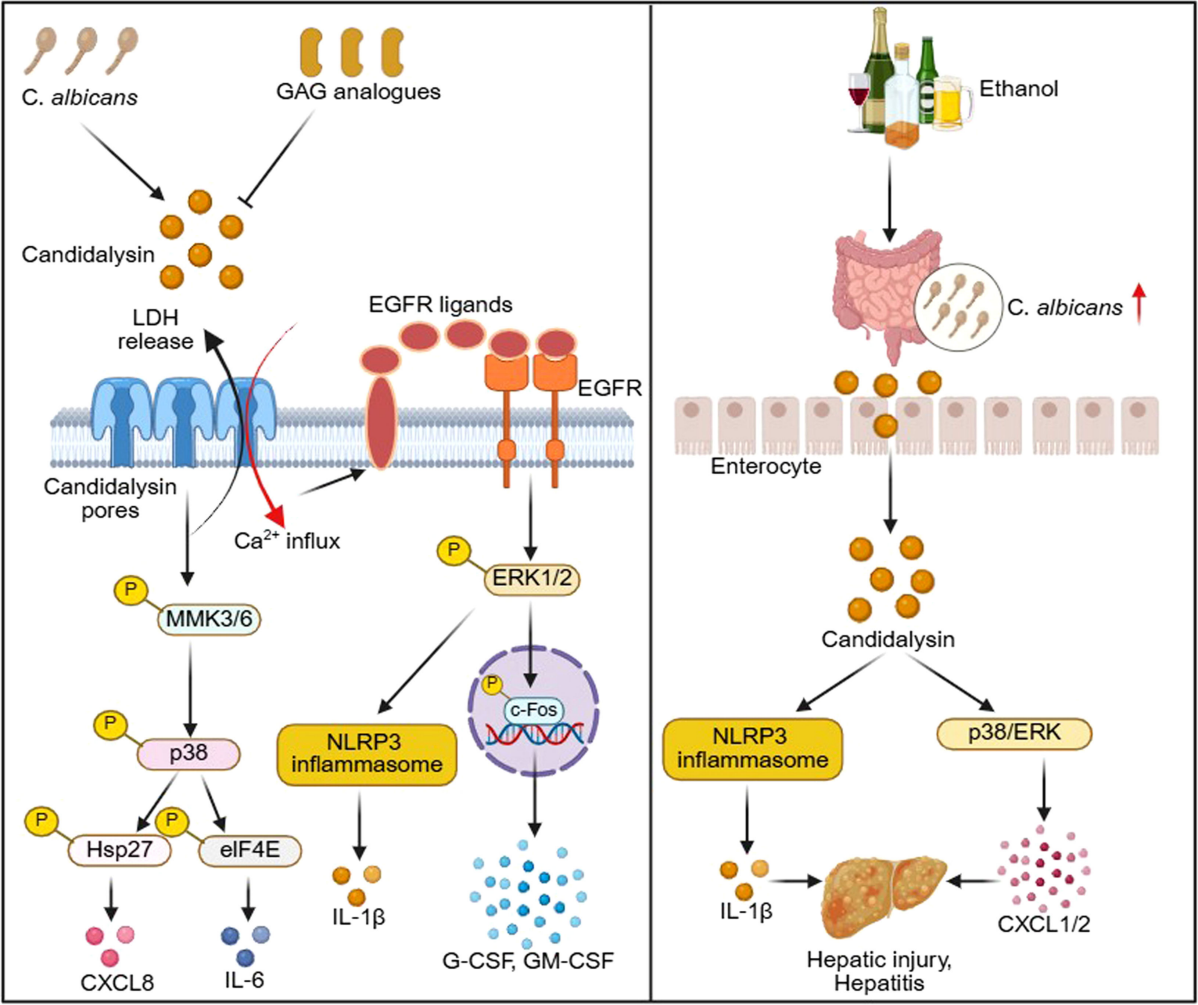
The candidalysin secreted by *C. albicans*, candidalysin interacts with the cell membrane to form pore-like structures that results in LDH release and calcium influx, which leads to the release of ligands for EGFR, ultimately resulting in the activation of EGFR, further activating the ERK1/2 pathway, promoting the phosphorylation of c-Fos. c-Fos enters the nucleus to initiate the expression of related genes, ultimately triggering the production of inflammatory mediators such as G-CSF and GM-CSF, and the activation of the NLRP3 inflammasome leads to the release of IL-1β. Through parallel pathways, candidalysin also activates p38, leading to the release of IL-6. In particular, candidalysin activates the MAPK pathway in endothelial cells and secretes CXCL-8. These inflammatory mediators can recruit phagocytes to the infection site, enhance the bactericidal activity, and initiate the innate immune response. In addition, excessive ethanol intake can lead to dysbiosis of the intestinal fungal flora, with an increase in the abundance of *C. albicans*. The candidalysin enter the liver through the portal vein via the damaged intestinal barrier, inducing the production of IL-1β and CXCL1/2, and promoting hepatic injury and hepatitis.

Candidalysin, a secreted cytolytic peptide toxin, is a key pathogenic factor of *C. albicans* (Moyes et al., 2016). This enzyme is synthesized as a proprotein by the ECE1 gene and the mature 31-amino-acid candidalysin is released from the hyphae of *C. albicans* (Naglik et al., 2019), forming pore-like structures on the host cell membrane, leading to calcium influx and cell damage (Westman et al., 2022; Russell et al., 2023), which leads to the release of ligands for EGFR, stimulates the innate immune response of epithelial cells by activating EGFR (Ho et al., 2019). EGFR activation leads to induction of MAPK signaling (via p38, ERK1/2) and the activation of c-Fos (Naglik et al., 2019), ultimately triggering the production of inflammatory mediators such as G-CSF and GM-CSF (Nikou et al., 2022), and the activation of the NLRP3 inflammasome leads to the release of IL-1β (Rogiers et al., 2019). Through a parallel pathway, candidalysin also activates p38, resulting in IL-6 release and Hsp27 phosphorylation (Nikou et al., 2022). In addition, candidalysin activates the MAPK pathway in endothelial cells and secretes CXCL8 (Swidergall et al., 2019). The research has found that the glycosaminoglycan (GAG) biosynthesis genes *XYLT2*, *B3GALT6*, and *B3GALT3* are crucial for the sensitivity of candidalysin (Lin et al., 2024). The absence of GAG leads to a stronger resistance of epithelial cells to the damage caused by candidalysin and *C. albicans* (Lin et al., 2024). Exogenous GAG or GAG analogues, such as dextran glucan sulfate, bind to candidalysin and inhibit its activity. In a mouse model of VVC, vaginal administration of dextran sulfate glucan sulfate significantly reduced epithelial cell damage, IL-1β release, and neutrophil aggregation (Lin et al., 2024). Host GAGs facilitate the activity of candidalysin, and GAG analogues can be used therapeutically to protect host cells from candidalysin-induced damage (Lin et al., 2024). Recent studies have shown that it has been found that candidalysin produced by *C. albicans* in the intestine can aggravate liver diseases caused by ethanol and is associated with an increase in mouse mortality (Chu et al., 2020). During the symbiotic growth period, studying the function of candidalysin in the host’s body while the intestinal microbiota is present will help reveal the mechanism of action of this toxin (**Figure 2**).

The core characteristics and mechanisms of candidalysin are well-defined: it exerts cytotoxicity by membrane insertion/lytication, activates pathways such as EGFR-MAPK and NLRP3 inflammasome to induce inflammation and damage in host cells, and targeted anti-infection strategies against this toxin have been summarized (Lortal et al., 2025). Recent studies in mice have further revealed its crucial role in colonization in different host sites, enriching functional understanding. In oral colonization, Candidalysin is a core element (Frois-Martins et al., 2025). Low virulence strains express ECE1 transiently when in contact with keratinocytes to synthesize this toxin. Mutant *ECE1* strains cannot invade the terminal differentiated epithelial layer of the mouse oral cavity and are difficult to evade the immune defense mediated by IL-17. The strict regulation of ECE1 can avoid excessive host damage and help *C. albicans* build an oral mucosal ecological niche, balancing colonization ability with host compatibility. In intestinal colonization, the function of Candidalysin has expanded to symbiotic regulation (Liang et al., 2024). When the microbiota is abundant, it enhances colonization adaptability by inhibiting competitive bacteria, and later, intestinal IgA targets the fungal hyphae to form negative selection pressure. This positive-negative balance makes it a key driver of intestinal symbiosis, rewriting the traditional notion that fungal hyphae are unfavorable for intestinal colonization.

## Virulence and pathogenic characteristics of *C. albicans*

*C. albicans*, as a typical opportunistic pathogenic fungus, possesses a strong pathogenicity due to the coordinated regulation of multiple virulence traits, which collectively support its transformation from a commensal to a pathogenic state. Under normal physiological conditions, it coexists with the host in a yeast form. However, when the intestinal flora is imbalanced or the host’s immune function is compromised, its morphological plasticity enables it to switch from the yeast form to the hyphal form, thereby breaking through the intestinal epithelial barrier and causing local mucosal infections or even systemic invasive diseases (Alonso-Monge et al., 2024). Additionally, it can precisely respond to host microenvironmental signals through phenotypic conversions such as white-opaque switching, dynamically regulating adhesion, colonization, immune evasion, and pathogenicity (Soll, 2014). The adhesin family (Als1, Als3, Hwp1) is a key molecular determinant of its pathogenicity. Among them, Als3, as a hypha-specific surface protein, can bind to host cadherins to induce endocytosis, mediate adhesion and colonization, invasion, and participate in iron metabolism to enhance pathogenicity (Liu and Filler, 2011). Als1 mainly promotes the adhesion of fungi to bacteria and the formation of mixed biofilms, while Hwp1 regulates the structural stability and cariogenic virulence of mixed biofilms by influencing hyphal morphology and extracellular polysaccharide production. Together, they shape the pathogenicity of the oral multi-microbial community (Martorano-Fernandes et al., 2023). The aspartic proteases secreted by *C. albicans* enhance fungal virulence by degrading host proteins, disrupting the epithelial barrier, and regulating immune responses (Naglik et al., 2003). The sophisticated regulation of cell wall components also contributes to its pathogenicity. The outer layer of mannan can mask β-glucan, reducing host innate immune recognition and inflammatory responses. Some strains can also enhance immune evasion through β-glucan modification (Selisana et al., 2024). The synergistic action of cell wall proteins (such as Hwp1, Als3) and mannan can mediate the adhesion of fungi to host epithelial cells and extracellular matrix (Mayer et al., 2013), while the dynamic remodeling of β-glucan and chitin supports hyphal extension and tissue penetration (Staniszewska, 2020). Moreover, *C. albicans* has excellent stress tolerance and environmental adaptability. Through antioxidant enzyme systems such as Sod/Cat and stress pathways such as Hog1/TOR, it coordinates morphological transitions and metabolic remodeling to resist host immune killing and tolerate microenvironmental stress (Davis, 2003). Additionally, its well-developed nutrient acquisition system, through nutrient sensing pathways such as TOR and Ras-cAMP-PKA, regulates the secretion of iron carriers, carbon source metabolism conversion, and hyphal morphogenesis, efficiently scavenging key nutrients such as iron, nitrogen, and carbon from the host to provide core support for colonization, invasion, and virulence expression (Fleck et al., 2011). The interplay of these virulence traits collectively constitutes the core mechanism of *C. albicans* pathogenicity.

## cAMP/PKA pathway

The transformation of *C. albicans* yeast into hypha is regulated by multiple pathways, among which the cyclic adenosine monophosphate (cAMP)/protein kinase A (PKA) pathway plays a major role. The core component of the cAMP/PKA pathway is Cyr1, which is the only adenylyl cyclase in *C. albicans* and can catalyze the synthesis of cAMP (Zeng et al., 2022). Targeted regulation of the Ras1-cAMP-PKA signaling pathway has inhibitory effects on the transformation of yeast into hypha and the formation of biofilms *in vitro*, and reduces the fungal load in a mouse systemic fungal infection model (Wang et al., 2024). *C. albicans* can transform from a symbiotic state to a pathogenic state, which may occur under conditions of host immune dysfunction, dysbiosis of the colonizing microbiota, or damage to the intestinal mucosal barrier (Zhai et al., 2020). *C. albicans* can invade host epithelial cells through either inducible endocytosis or active penetration (da Silva Dantas et al., 2016). Inducible endocytosis is mainly achieved through two invasive proteins, ALS3 and SSA1, which achieve this by binding to the N-cadherin and E-cadherin of host endothelial cells and epithelial cells (Sun et al., 2010). However, in the process of active penetration, the cAMP/PKA signaling pathway activates the expression of hypha-related genes through Efg1, a process that is considered necessary for active penetration and epithelial cell damage (Rai et al., 2021). Study has found that *Sodium New Houttuyfonate* inhibits the formation of *C. albicans* biofilms and fungal infection in *Galleria mellonella* larvae by regulating the Ras1-cAMP-Efg1 pathway (Wu et al., 2020c).

## Strain-specific heterogeneity shapes pathogenicity, biofilm traits and drug susceptibility in *C. albicans*

*C. albicans* exhibits remarkable intraspecific strain heterogeneity, which is driven by genetic variations including aneuploidy, loss of heterozygosity, copy number variations and single nucleotide polymorphisms, as well as epigenetic phenotypic switching and niche-specific adaptive regulation, and this heterogeneity directly leads to significant differences in pathogenicity, biofilm formation ability and drug responsiveness among different strains (Hirakawa et al., 2015). In terms of pathogenicity, distinct strains show obvious divergence in core virulence traits: for example, chromosome 7 trisomic strains present attenuated hyphal formation due to NRG1 overexpression, while euploid strains form robust hyphae with stronger tissue adhesion and invasion capabilities (Kakade et al., 2023). For biofilm formation, there are significant differences in biomass, structural density and matrix composition among strains: high biofilm-forming strains upregulate the expression of ALS3, HWP1 and BCR1 genes to produce dense and stable biofilms with abundant extracellular matrix (Nobile et al., 2012), while chromosome 7 trisomic strains show drastically reduced biofilm biomass and fragile structural characteristics (Mishra et al., 2025), such strain-specific biofilm differences are closely associated with persistent colonization of clinical medical devices and mucosal surfaces. In terms of drug responsiveness, different *C. albicans* strains show highly variable tolerance and resistance to common antifungal agents. Some clinical isolates from patients with head and neck cancer exhibit moderate to high fluconazole tolerance, with high-tolerance strains upregulating *ERG11*, *CDR1* and other genes to maintain biofilm integrity and drug survival under antifungal pressure (Hatami et al., 2023). Chromosome 5 trisomy is the dominant mechanism for *Candida parapsilosis* to adapt to caspofungin, replacing rare *FKS* gene mutations as the primary driver of echinocandin tolerance; crucially, this aneuploidy also confers cross-tolerance to unrelated 5-flucytosine, with instability of aneuploidy leading to reversible drug tolerance (Sun et al., 2023b).Collectively, the intraspecific differences of *C. albicans* are key biological characteristics affecting its clinical infection outcomes, treatment efficacy and recurrence risk, and fully considering such strain heterogeneity is of great significance for optimizing antifungal therapy and improving the management of refractory candidiasis.

## Interaction between *Candida* and bacteria

*C. albicans* has complex dynamic interactions with intestinal and oral bacteria, mainly divided into two modes: competitive inhibition and synergistic enhancement. The balance of these interactions is crucial for the stability of the host’s microecology and the progression of diseases. In the intestinal microecology, the relationship between *C. albicans* and bacteria is characterized by “competition-balance”. *C. albicans* can inhibit the growth of competing bacteria through the filament toxin Candidalysin, enhancing its colonization fitness in a sufficient microbial environment. Later, intestinal IgA targeting the filaments creates negative selection pressure, making Candidalysin a key driver of intestinal symbiosis and overturning the traditional view that filaments are unfavorable for intestinal colonization (Liang et al., 2024). Conversely, bacteria can also regulate *C. albicans*. For instance, the secretions of Escherichia coli biofilms can inhibit the formation of *C. albicans* biofilms and filament development by regulating the activities of filament genes (*ECE1*, *HWP1*) and transcription factors (*NRG1*, *EFG1*) (Bandara et al., 2013). In antibiotic intervention models, when the cecal microbiota is reconstructed, bacteria limit the excessive proliferation of fungi through nutritional and spatial competition, while fungal colonization also affects the composition and recovery of the bacterial microbiota, maintaining the stability of the intestinal microecology (Mason et al., 2012). In the oral microecology, the relationship between *C. albicans* and bacteria is mainly characterized by “synergistic enhancement”, promoting the progression of oral diseases. Rat models have confirmed that co-culturing *C. albicans* with Enterococcus faecalis enhances the virulence of mixed biofilms, increases drug resistance and invasiveness, and aggravates periapical tissue damage (Du et al., 2021). The mechanism involves mutual regulation of virulence factors, *E. faecalis* promotes the formation of fungal filaments and the release of Candidalysin, while the fungus enhances the activity of *E. faecalis* virulence factors (such as CylLL-hemolysin), synergistically exacerbating host cell damage (Kapitan et al., 2025). Such synergistic effects are not limited to the oral cavity; for example, co-infection of Proteus mirabilis and *C. albicans* can synergistically aggravate intestinal epithelial cell damage, with the mechanism being the combined action of the urease of the former and the Candidalysin of the latter, enhancing fungal invasiveness and host cell death (Niemiec et al., 2022).

Individuals with irritable bowel syndrome (IBS) exhibit elevated levels of bacteria related to intestinal inflammation, such as *Enterobacteriaceae* and *Streptococcus*, while the content of beneficial bacteria (such as *Faecal Streptococcus*) decreases (Sciavilla et al., 2021). IBS is also associated with fungal microbiota imbalance, especially overgrowth of *Candida* (Das et al., 2021). Additionally, in patients with inflammatory bowel diseases (such as Crohn’s disease and ulcerative colitis), the expansion of the commensal bacterium *Akkermansia muciniphila* can exacerbate the colonization of *Candida tropicalis* (Di Martino et al., 2022). The abundance of *Escherichia coli* in the gut significantly affects the hyphae and biofilms formation of *Candida*, and upregulates pathogenic genes such as *ALS3* and *HWP1* (Farrokhi et al., 2021). The proliferation of adherent-invasive *Escherichia coli* is associated with the occurrence of Crohn’s disease, and it can promote the hyphae invasion of *Candida* into intestinal epithelium and lamina propria through the ability to destroy and invade epithelial cells and trigger intestinal inflammation (Zhilu et al., 2021; Kittana et al., 2023). Due to changes in the microbial community and metabolome, the colonization of *C. albicans* in mice treated with antibiotics increased (Gutierrez et al., 2020). Compared with the cecal metabolome of untreated mice, the levels of primary bile acids (especially taurocholic acid (TCA)), carbohydrates, and sugar alcohols were elevated, while the levels of bacterial metabolites (secondary bile acids and carboxylic acids) were decreased (Gutierrez et al., 2020). TCA was identified as the main bile acid regulating the colonization and spread of *C. albicans* in the intestinal tract (Thangamani et al., 2021). Mice drinking water containing TCA had their commensal microbial community disrupted, with significant reductions in the numbers of *Lactobacillus johnsonii*, *Streptococcus faecalis*, and *Clostridium* sp*ecies* (Thangamani et al., 2021). Elevated TCA levels also altered the innate (neutrophils and macrophages) and adaptive (Th1 and Th17 cells) immune responses in the mucosa, promoting the colonization of *C. albicans* in the intestinal tract (Datta et al., 2022). In addition, beta-lactam antibiotics can cause intestinal bacteria to release a “peptidoglycan storm”, facilitating the transformation of *C. albicans* yeast into hypha, and subsequently leading to systemic spread (Tan et al., 2021).

In the colitis mouse model induced by dextran sulfate sodium (DSS), compared with mice infected with *C. albicans* alone, mice infected with both *C. albicans* and bacteria had milder colitis symptoms and lower mucosal fungal load (Mao et al., 2021). Additionally, *Faecalibacterium prausnitzii* and its supernatant can promote the activity of the inflammasome containing Nod-like receptor protein 6, increase the secretion of IL-1β, IL-18 and AMPs, thereby resisting *C. albicans* (Mao et al., 2021). The gut microbiota produces various metabolites, especially short-chain fatty acids (SCFAs), which can activate the mucosal immune system and induce AMPs production, thereby combating pathogens (Liu et al., 2023). *Bacteroides thetaiotaomicron* and *Lactobacillus johnsonii* generate fatty acids, particularly oleic acid and palmitic acid, regulate inflammatory immune responses, and eliminate *Candida glabrata* in the DSS-induced colitis mouse model (Charlet et al., 2022). Therefore, by exploring the relationship between the bacterial-fungal interactions and the regulation of host immunity, this provides the possibility for the development of novel antifungal strategies.

## Current antifungal drugs

Currently, the first-line clinical antifungal drugs include polyenes (e.g., amphotericin B), azoles (e.g., fluconazole) and echinocandins (e.g., caspofungin) (Wiederhold, 2017; Somer et al., 2011). Polyenes bind to fungal cell membrane ergosterol, leading to cell lysis and are used for severe infections. Azoles inhibit ergosterol biosynthesis and demonstrate excellent curative effects in mild-to-moderate mucosal infections (vulvovaginal, oral) with high oral bioavailability and good tolerance. Echinocandins inhibit the fungal (1,3)-β-D-glucan cell wall biosynthesis and low toxicity, serving as the first-line drugs for invasive infections in critically ill patients (Pappas et al., 2016). Some traditional antifungal drugs, such as itraconazole, voriconazole, and amphotericin B, have serious toxicities (Zavrel and White, 2015). However, these mainstream therapies face critical limitations including the rapid emergence of drug resistance, FKS1/FKS2 gene mutations reducing β-1,3-glucan synthase affinity, Targeted enzyme (Erg11p) modification, overexpression of efflux pumps (CDR1, MDR1), and upregulation of the ergosterol pathway mediate azole resistance (Pristov and Ghannoum, 2019). Poor penetration of drugs into fungal biofilms leading to persistent and recurrent infections (Desai et al., 2014), and unsatisfactory therapeutic responses in immunocompromised patients with CARD9 deficiency (Zhang et al., 2023). Due to the extensive use of antifungal drugs, the resistance of *C. albicans* is increasing, which poses a serious threat to antifungal therapy. Therefore, there is an urgent need to explore effective antifungal drugs with novel drug targets to address the challenges faced in the antifungal field.

## Emerging treatment for *C. albicans*

Emerging therapeutic approaches show great translational potential for overcoming existing bottlenecks. Beneficial gut bacteria (e.g., *Lactobacillus*) inhibit fungal overgrowth and pathogenicity via nutrient competition and metabolite secretion to maintain host homeostasis, while dysregulated bacteria can break the mucosal barrier and synergize with fungi to promote invasive infection (Song et al., 2025). *C. albicans* is the primary commensal fungus that primes human anti-fungal Th17 responses, and cross-reactive Th17 cells induced by it are critical for both protective immunity against other fungi and the pathogenesis of fungal-related inflammatory diseases. It demonstrates that these cross-reactive Th17 cells, activated by shared fungal antigens (e.g., from *Aspergillus fumigatus*), defend against diverse fungal infections but can also drive pathological inflammation in conditions like allergic bronchopulmonary aspergillosis. Thus, the IL-17 agonist, a promising immunomodulator, represents a valuable candidate for the development of novel antifungal agents (Bacher et al., 2019). A recent study designed a “nano-shield” hydrogel co-delivering myricetin-laurate nanoparticles, which exerts antifungal effects by disrupting fungal membranes and biofilms while accelerating skin regeneration via promoting fibroblast proliferation and collagen deposition, showing promising therapeutic potential for refractory fungal skin infections (Zhang et al., 2025).

In addition, there are other emerging antifungal treatments, they include monoclonal antibodies (such as C3.1) and immunomodulators (such as TLR9 agonists). Monoclonal antibodies target fungal cell wall epitopes and enhance phagocytosis, suitable for drug resistant strains. Immunomodulators activate innate immune signaling and assist in the treatment of invasive infections in immunocompromised patients. The new generation of azoles (such as isavuconazole) broaden the antifungal spectrum. biofilm disruptors (such as caspofungin combined with quorum sensing inhibitors) can interfere with biofilm formation and are used for refractory mucosal infections. Recombinant antigen vaccines (such as Als3p) induce humoral/cellular immunity and are used for prevention in high-risk populations. The attenuated *C. albicans* vaccines stimulate immunity through live strains and prevent recurrent mucosal infections (**Table 2**).

**Table 2:** Summary of current and emerging therapeutic strategies for *Candida albicans* infections.

Therapeutic category	Agents/approaches	Mechanism of action	Clinical applications	References
Classic Antifungals	Azoles (Fluconazole, Voriconazole)	Inhibit ergosterol synthesis by targeting lanosterol 14α-demethylase	Mucosal candidiasis (e.g., vulvovaginal candidiasis), invasive candidiasis prophylaxis	(Bodey, 1992) (Benitez and Carver, 2019)
	Echinocandins (Caspofungin, Micafungin)	Inhibit β-1,3-glucan synthesis, disrupting fungal cell wall integrity	Invasive candidiasis (first-line for critically ill patients), refractory infections	(Szymanski et al., 2022)
	Polyenes (Amphotericin B)	Bind to ergosterol, forming membrane pores and inducing fungal cell lysis	Severe invasive candidiasis, refractory mucosal infections	(Hamill, 2013) (Wang et al., 2021)
Emerging Immune-based Therapies	Monoclonal Antibodies (C3.1, 9F2)	Target fungal cell wall epitopes (β-1,2-mannotriose, phosphoglycerate kinase 1); enhance phagocytosis and disrupt biofilms	Invasive candidiasis (especially multidrug-resistant strains like *C. auris*)	(Rosario-Colon et al., 2021)
	Immunomodulators (TLR9 agonists, Dectin-1 activators)	Activate innate immune signaling (NF-κB pathway) upon fungal β-1,3 glucan recognition, thereby enhance macrophage function	Adjunct therapy for immunocompromised patients with invasive candidiasis	(Khan et al., 2016)
Novel Antifungal Agents	Next-generation Azoles (Isavuconazole, Posaconazole)	Improved ergosterol synthesis inhibition; broader spectrum against non-albicans Candida	Invasive candidiasis, prophylaxis in immunocompromised hosts (e.g., transplant patients)	(Lewis et al., 2022)
	Biofilm Disruptors (Caspofungin + Quorum-sensing inhibitors)	Disrupt fungal biofilm formation; enhance penetration of antifungals into biofilm matrix	Refractory mucosal candidiasis (e.g., recurrent vulvovaginal candidiasis)	(Su et al., 2022) (Mehmood et al., 2019)
Vaccine Developments	Recombinant Antigen Vaccines (Als3p, Hwp1)	Induce humoral (antibody) and cellular (T cell) immunity against fungal adhesins	Prophylaxis in high-risk populations (e.g., ICU patients, immunocompromised hosts)	(Singh et al., 2025)
	Attenuated C. albicans Vaccines	Stimulate innate and adaptive immunity via live-attenuated fungal cells	Prophylaxis for recurrent mucosal candidiasis	(Wang et al., 2015) (Kaur et al., 2023)

*C. albicans* is the main cause of VVC, a common gynecological disease. Approximately 75% of women of childbearing age will experience at least one infection in their lifetime, and 5% to 8% of them will develop recurrent VVC. Symptoms include a burning sensation, pain, and excessive vaginal discharge, which significantly affect the quality of life (Spaggiari et al., 2022). In recent years, probiotics have emerged as one of the candidate alternatives to antibiotics. *Lactobacillus* is the dominant probiotic in the healthy vaginal microbiota of women, that can resist various pathogens, such as *Candida* species, and help enhance resistance to infections (Spaggiari et al., 2022). *Lactobacillus* inhibit the VVC by producing lactic acid and bacteriocins (Zangl et al., 2019). Additionally, proanthocyanidins in cranberries can enhance the antifungal activity of probiotics, effectively inhibiting the colonization and biofilm formation of *C. albicans*, providing a highly promising alternative therapy strategy for the treatment of VVC (Wu et al., 2025). Probiotics and fecal microbiota transplantation (FMT) have shown certain efficacy in combating *Candida* infections (Li et al., 2022; Leonardi et al., 2020). Probiotic genera such as *Bacillus*, *Bifidobacterium*, *Lactobacillus*, *Saccharomyces*, and *Metschnikowia* can inhibit *C. albicans* growth (Chow et al., 2024). Respondents with *Clostridiodes difficile* infection had a higher fungal diversity after FMT, including more species such as *Saccharomyces*, *Aspergillus*, and *Penicillium* species (Zuo et al., 2018). Prostaglandins (PG) produced by bacteria can promote the hyphal growth of *C. albicans* by activating Cyr1p (Xu et al., 2008). Monoclonal antibodies targeting the conserved PG portion (N-acetyl-L-isoglutamine) have been developed, which can specifically bind to PG (Huang et al., 2019). This antibody can effectively prevent the hyphal growth of *C. albicans* and can neutralize the circulating PG in mice, indicating its potential application value in treating *C. albicans* infections (Huang et al., 2019).

There have also been new findings regarding targeted therapy against *C. albicans*. Inhibitors targeting the member Yck2 of the casein kinase 1 family have shown certain single-agent activity against *C. albicans* that is resistant to echinocandin antifungal drugs, and can enhance the therapeutic effect of echinocandin antifungal drugs (Puumala et al., 2025). It was discovered that Sdd3 negatively regulates the upstream activating factor Rho1 GTPase through direct interaction with the GTPase-activating protein Bem2 of Rho1, thereby significantly reducing the chitin content in the fungal cell wall and causing defects in biofilm formation. Sdd3 may serve as an antifungal target for biofilm infections (Pang et al., 2025). Panthothenate kinase (PanK) is responsible for converting panthothenate into coenzyme A (CoA), which is crucial for the survival of fungi. The effectiveness of targeting PanK with small molecule inhibitors as a strategy for developing effective antifungal therapies has been found (Regan et al., 2025). The RNA polymerase-related factor 1 complex (Paf1C) has gradually become a key transcriptional regulatory factor in eukaryotes (Francette et al., 2021). This complex consists of core subunits (*Paf1*, *Leo1*, *Cdc73*, *Rtf1*, and *Ctr9*) and a unique subunit specific to humans (*Ski8*). It affects chromatin structure and transcription accuracy through epigenetic modifications (Park et al., 2023). The deletion of *Ctr9* affects the proliferation of *C. albicans* cells, hyphal formation, and methionine metabolism, thereby significantly weakening the pathogenicity of *C. albicans* (Park et al., 2024).

**Figure 3:**
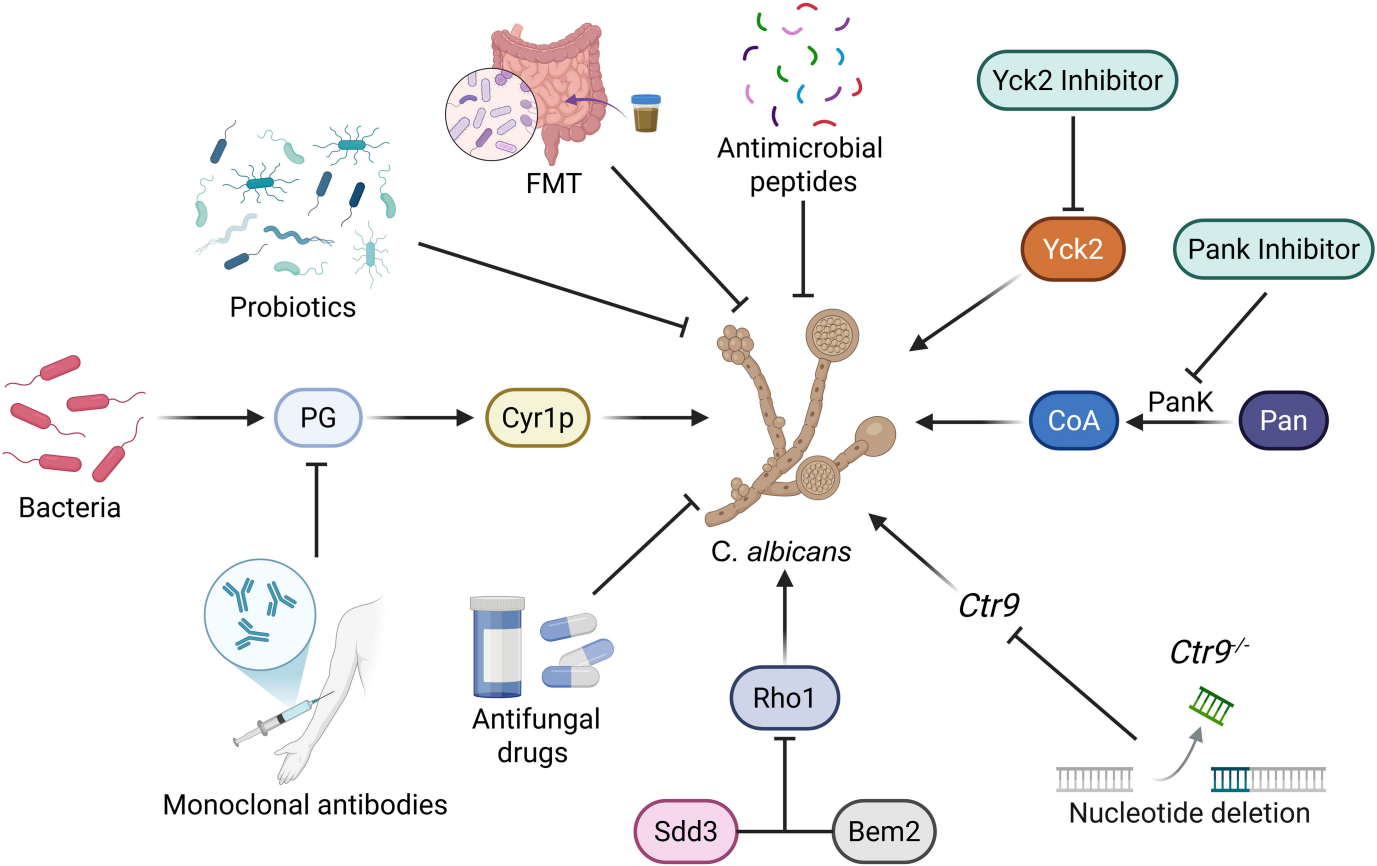
For the treatment of *C. albicans*, in addition to traditional antifungal drugs, there are also probiotic intervention, fecal microbiota transplantation, antibacterial peptides, as well as targeted small molecule inhibitors or monoclonal antibodies against the key biological processes of *C. albicans*, or deletion of a certain key virulence gene to exert antifungal effects.

The microbial community and the molecules release can activate the mucosal immune system and promote the production of AMPs (Wang et al., 2023). Fungi can also trigger host immune responses, including the production of AMPs and inflammatory cytokines (Netea et al., 2015). Many AMPs with antifungal activity, immunomodulatory molecules produced by the microbial community, the strong hyphal induction activity of PG, genetic factors that cause the adaptation and morphological changes of *C. albicans* in the host body, and potential probiotics, have opened up numerous avenues for developing novel antifungal strategies (Chow et al., 2024). Such as, the antimicrobial peptide LL-37 can kill *C. albicans* by targeting the cell wall and plasma membrane, thereby causing cell cycle arrest (Rather et al., 2022). The PYY1–36 secreted by paneth cells in the intestinal epithelium specifically targets the hyphae cells of *C. albicans* and kills this fungus by destroying the cell membrane, but has no such effect on yeast-shaped fungi (Pierre et al., 2023). The whole housefly larvae insect SVWC peptide 1 isolated from *Housefly* larvae was found to inhibit the invasion of *C. albicans* into epithelial cells by affecting hyphal formation and the expression of genes related to adhesion factors (Chen et al., 2024). Such strategies may involve direct intervention against pathogens, utilization of the host immune system, and regulation of the composition of the host microbial community, provides potential drug candidates for the treatment of *C. albicans* infections (**Figure 3**).

## Future perspectives and challenges

In future research, the primary focus should be on deciphering the precise molecular mechanisms underlying *C. albicans*-mediated regulation of inflammatory, metabolic, and signaling pathways, as well as clarifying its complex interplay with the host, including the key molecular targets involved in immune evasion, microenvironment remodeling, and genotoxicity. Despite advances in antifungal monotherapy and combination strategies, critical challenges remain, such as overcoming the escalating drug resistance, optimizing individualized therapeutic regimens tailored to diverse patient populations, and improving long-term treatment efficacy to reduce recurrence and disease progression. To address these gaps, multidisciplinary collaborative efforts integrating molecular biology, immunology, pharmacology, and clinical medicine are essential to fully unravel the pathogenic roles of *C. albicans* in various diseases, which will facilitate the identification of novel therapeutic and preventive targets and the development of more effective, safe, and durable intervention strategies against *C. albicans*-associated disorders.

## Glossary

ALS3: Agglutinin - like sequence protein 3
AMPs: Antimicrobial peptides*;* B3GALT, beta-1,3-galactosyltransferase
Bcl10: B-cell lymphoma/leukemia 10
*C. albicans*: *Candida albicans*
cAMP: Cyclic adenosine monophosphate
CARD9: Caspase recruitment domain-containing protein 9
Cdc73: Cell division cycle 73
CDR1: Candida drug resistance 1
c-Fos: cellular FBJ osteosarcoma oncogene
cGAS: Cyclic GMP-AMP synthase
CLRs: C-type lectin receptors
CMC: Cutaneous and mucosal candidiasis
CoA: Coenzyme A
Csh1p: Cell surface hydrophobicity protein 1
Ctr9: CTR9 homolog, Paf1/RNA polymerase II complex component
CXCL: C-X-C motif chemokine ligand
Cyr1: Adenylate cyclase
DCs: Dendritic cells
Dectin-1: C-type lectin domain family 7 member A
DSS: Dextran sulfate sodium
ECE1: Cell elongation protein 1
EFG1: Enhanced filamentation 1
ERK: Extracellular signal-Regulated Kinase
ERG11: Lanosterol 14α
FKS: FKS glucan synthase subunit
FMT: Fecal microbiota transplantation
GAG: Glycosaminoglycan
GM-CSF: Granulocyte-macrophage colony-stimulating factor
H3K56ac: Histone H3 lysine 56 acetylation
HATs: Histone acetyltransferases
HIF-1α: Hypoxia-inducible factor 1 alpha
HDACs: Histone deacetylases
HOG: High Osmolarity Glycerol
HWP1: Hyphal wall protein 1
IBS: Irritable bowel syndrome
Leo1: Leo1 component of Paf1/RNA polymerase II complex
IFN-I: Type I interferon
IL: Interleukin
IRF: Interferon Regulatory Factor
JEN1: Jen1-type carboxylate transporter 1
JEN2: Jen2-type carboxylate transporter 1
MALT1: Mucosa-associated lymphoid tissue lymphoma translocation protein 1
MAPK: Mitogen-activated protein kinase
MDR1: Multidrug resistance 1
mTOR: Mammalian target of rapamycin
MUC2: Mucin 2
NF-κB: Nuclear Factor-kappa B
NLRs: Nucleotide-binding oligomerization domain (NOD)-like receptors
NLRP3: NOD-like Receptor Family Pyrin Domain Containing 3
NRG1: Nitrogen-regulated gene 1
Paf1C: Polymerase- associated factor 1 complex
PAMPs: Pathogen-associated molecular patterns
PD-L1: Programmed cell death ligand 1
PG: Prostaglandins
PanK: Panthothenate kinase
PKA: Protein kinase A
RBF1: RPG - box binding factor 1
Rtf1: Regulation of transcription factor 1
RVVC: Recurrent vulvovaginal candidiasis
SCFAs: Short-chain fatty acids
SSA1: Heat shock 70 - kDa protein 1
STING: Stimulator of interferon genes
Syk: Spleen tyrosine kinase
TBK1: TANK-binding kinase 1
TCA: Taurocholic acid
Th17: T helper 17 cell
TLRs: Toll-like receptors
TNF-α: Tumor Necrosis Factor α
TUP1: Transcriptional repressor protein 1
VVC: Vulvovaginal candidiasis
XYLT2: Xylosyltransferase 2
